# Draft genome sequences of six bacterial strains isolated from Cannabis rhizosphere soil

**DOI:** 10.1128/MRA.00653-23

**Published:** 2023-10-31

**Authors:** Tyson Bookout, Kira L. Goff, Jeff Gauthier, Roger C. Levesque, Shawn Lewenza

**Affiliations:** 1 Department of Microbiology, Immunology and Infectious Diseases, University of Calgary, Alberta, Canada; 2 Faculty of Science and Technology, Athabasca University, Athabasca, Alberta, Canada; 3 Institute of Integrative and Systems Biology, Laval University, Quebec, Canada; Indiana University, Bloomington, Bloomington, Indiana, USA

**Keywords:** *Pseudomonas*, *Cannabis sativa*, rhizosphere, soil microbiology

## Abstract

Although bacterial isolates from Cannabis flowers were reported and sequenced, few from its rhizosphere have been characterized. Here we report the draft genomes of six bacterial strains isolated from Cannabis rhizosphere soil samples. These sequences may shed light on plant-microbe interactions in the Cannabis rhizosphere at the molecular level.

## ANNOUNCEMENT

The Cannabis plant has been cultivated for its nutritional value, durable fibers, medicinal use, and recreation for thousands of years (1). The ratio of cannabidiol (CBD) and tetrahydrocannabinol (THC) is important for both medicinal and recreational use, and the yields of these metabolites could be impacted by the microbial communities associated with the cannabis plant (2). Although bacterial isolates have been recovered and sequenced from Cannabis flowers (3), few have been reported from the Cannabis rhizosphere. Such bacterial isolates may have applications in biotechnology or agriculture.

Here we present the draft genome sequences of six bacterial isolates (Table 1) obtained from the rhizosphere of cultivated Cannabis plants (Anandia Labs; Vancouver, Canada). One gram of soil, recovered from Cannabis roots, was suspended in 10 mL of double-distilled water. The soil particulates were sedimented by centrifugation and the soil water extract spread-plated on *Pseudomonas* isolation agar (PIA, BD Difco). After incubation at 37ºC, single colonies were isolated by three rounds of single-colony isolation with streaking, again on PIA agar.

**TABLE 1:** Main assembly metrics and number of features for each assembled genome^*a*^

Isolate ID	Organism	TYGS dDDH d4 (% and 95% CI)	Raw coverage (x)	Genome size (Mbp)	# Scaffolds	Scaffold N50 (bp)	Completeness	GC content (%)	# CDS	# tRNAs
SPPC 2814	*Pseudomonas sp*. CAN1	41.5 (39.0–44.0)^***b***^	33	6.81	99	638,296	100.0	65.7	6,221	116
SPPC 2815	*Pseudomonas citronellolis* CAN5	89.2 (85.9–91.9)	40	7.11	64	936,389	100.0	67.5	6,291	114
SPPC 2816	*Aeromonas caviae* CAN6	84.4 (81.7–86.8)	25	4.77	114	228,986	100.0	61.0	4,438	163
SPPC 2817	*Serratia bockelmannii* CAN8	87.8 (84.3–90.6)	27	5.23	55	1,162,636	99.7	59.4	4,953	136
SPPC 2818	*Pseudomonas rhodesiae* CAN13	80.3 (77.4–82.9)	44	5.91	43	898,063	99.7	60.3	5,414	105
SPPC 2819	*Variovorax sp*. CAN15	50.4 (47.8–53.0)^*b*^	30	6.92	193	401,761	100.0	67.2	6,602	98

^*a*^ dDDH d4: proportion of sequence identity matched between draft genome and the most similar reference genome. Raw coverage: average number of reads covering each nucleotide position in the assembly. Completeness: an estimate calculated by CheckM, based on the presence of core genes, and the consistency of nucleotide usage statistics across scaffolds. CDS: coding DNA sequences. tRNAs: transfer RNA genes.

^*b*^ Potential new species (dDDH with closest Type Strain Genome Server [TYGS] reference < 70%).

Brain-heart infusion (BHI) agar (BD Difco) was used to culture bacterial isolates at room temperature (~25⁰C) for 48 h. Single colonies were suspended in 2 mL BHI broth and grown to an OD_600_ of 1.0. Cells were pelleted by centrifugation (10,000× *g* for 20 min at 4⁰C) and genomic DNA was extracted using a DNeasy Blood & Tissue kit (QIAGEN) according to the recommended protocol for Gram-negative bacteria. DNA purity was confirmed using a NanoDrop 2000 spectrophotometer (Thermo Fisher) and quantified using a Qubit dsDNA BR kit (Thermo Fisher). The G-tube fragmentation spin-column protocol (Covaris) was used to shear 1 µg genomic DNA into 400–700 bp fragments. These were then sequenced in paired ends (2 × 300 bp) on a MiSeq apparatus (Illumina) using the KAPA HyperPrep kit (Roche Sequencing Solutions) with Illumina TruSeq 3-PE adapters and the manufacturer’s recommended protocol for bacterial WGS sequencing. Read quality was visualized with FastQC (version 0.11.8 (4)) and quality filtering/adapter removal was performed with TRIMMOMATIC (version 0.39–2 (5)), keeping sequences below Q15 quality score over a minimum read length of 36 bp. Trimmed Illumina reads were assembled with the A5 pipeline (version 20150522 (6)). Annotation was done *via* the NCBI Prokaryotic Genome Annotation Pipeline (PGAP) version 6.4 (7). Taxonomy was assigned to draft genomes with whole-genome *in silico* digital DNA-DNA hybridization (dDDH) *via* the TYGS (8). Genome completeness was evaluated with CheckM (9). All software was run with default parameters unless otherwise specified.

Our results include two potential new species with dDDH d4 below 70% (Table 1). Further genomic analysis of these strains will facilitate a greater understanding of the molecular mechanisms of plant-microbe interactions in the growth of the Cannabis plant and the potential impact of secondary metabolite production.

## Data Availability

These draft genome sequences have been deposited in DDBJ/ENA/GenBank BioProject accession PRJNA971652. Draft genome accession numbers are as follows: JASMRU000000000, JASMRV000000000, JASMRW000000000, JASMRX000000000, JASMRY000000000, and JASMRZ000000000, and raw reads were, respectively, deposited in the Sequence Read Archive under accessions: SRX20708416, SRX20708417, SRX20708418, SRX20708419, SRX20708420, and SRX20708421.
